# Targeting DYRKs in Cardiovascular Diseases: From Biological Mechanisms to Therapeutic Translation

**DOI:** 10.3390/ijms27083456

**Published:** 2026-04-12

**Authors:** Long Jiang, Siyu Peng, Junchen Ma, Tingfei Liu, Xiaoru Long, Lin Li, Huaying Wu

**Affiliations:** Key Laboratory of Study and Discovery of Small Targeted Molecules of Hunan Province, Hunan Normal University Health Science Center, Hunan Normal University, Changsha 410013, China; longjiang27@hunnu.edu.cn (L.J.); pengsiyu0106@hunnu.edu.cn (S.P.); 2990468446@hunnu.edu.cn (J.M.); liutingfei@hunnu.edu.cn (T.L.); longxiaoru@hunnu.edu.cn (X.L.)

**Keywords:** DYRKs, myocardial infarction, heart failure, cardiac fibrosis, kinase inhibitors, therapeutic translation

## Abstract

Cardiovascular diseases (CVDs) remain the leading cause of mortality globally, characterized by myocardial injury, pathological structural remodeling, and progressive deterioration of cardiac function. Clinical manifestations include post-infarct functional impairment, pathological cardiac hypertrophy, interstitial fibrosis, malignant arrhythmias, and end-stage heart failure. Although the dual-specificity tyrosine-regulated kinase (DYRK) family has been extensively investigated in cancer and neurodegenerative disorders, emerging evidence highlights DYRKs as critical upstream regulators in a wide spectrum of cardiovascular pathological processes. However, current research is largely confined to individual isoforms or isolated signaling pathways, lacking systematic integration of isoform-specific functions, dose- and spatiotemporal-dependent effects, as well as bidirectional regulatory roles in chronic cardiac remodeling. This review systematically summarizes the molecular mechanisms of the DYRK family across major cardiovascular disease models, with particular emphasis on the functional specificity of distinct DYRK isoforms and their translational potential as therapeutic targets. We further provide an integrated theoretical framework to facilitate the development of isoform-selective, context-dependent precision therapies for cardiovascular diseases.

## 1. Introduction

Cardiovascular diseases (CVDs) are a leading cause of death worldwide. Despite substantial differences in etiology and clinical presentation, most CVDs share several core pathological features, including limited cardiomyocyte proliferative capacity, dysregulated cell death and stress responses, persistent pathological hypertrophic signaling, and impaired mitochondrial and metabolic homeostasis. The coordinated disruption of these processes drives progressive cardiac remodeling and ultimately culminates in end-stage outcomes such as heart failure. Therefore, identifying key signaling molecules that regulate these interconnected pathological pathways is crucial for elucidating CVD pathogenesis and developing targeted therapies.

The dual-specificity tyrosine-regulated kinase (DYRK) family is an evolutionarily conserved group of protein kinases with both tyrosine (Tyr) and serine/threonine (Ser/Thr) kinase activities. A hallmark of DYRKs is their one-time co-translational autoactivation mechanism, in which intramolecular autophosphorylation during protein synthesis stabilizes the active conformation by phosphorylating a critical tyrosine residue within the activation-loop YxY motif (e.g., Tyr321 in DYRK1A), thereby generating a constitutively active catalytic unit [1]. This unique activation mode allows DYRKs to function independently of continuous upstream stimulation and may facilitate sustained signaling under cardiovascular stress conditions, such as pressure overload, ischemia/reperfusion injury, and metabolic disturbance.

Although the roles of DYRKs in CVDs have not been fully defined, accumulating evidence implicates this kinase family in myocardial infarction [2], cardiac hypertrophy [3], myocardial fibrosis [4], heart failure [5]. By regulating cell-cycle progression [6], the survival-apoptosis balance [7], stress signaling pathways [5], and mitochondrial metabolism [8], DYRKs may serve as a central signaling hub linking cardiac development, injury repair, and pathological remodeling. Given the limited regenerative capacity of postnatal cardiomyocytes, elucidating the molecular structure and regulatory mechanisms of DYRKs may provide important insights into CVD pathogenesis and therapeutic targeting.

## 2. The Molecular Function of DYRKs

### 2.1. Structure of DYRKs

**Figure 1:**
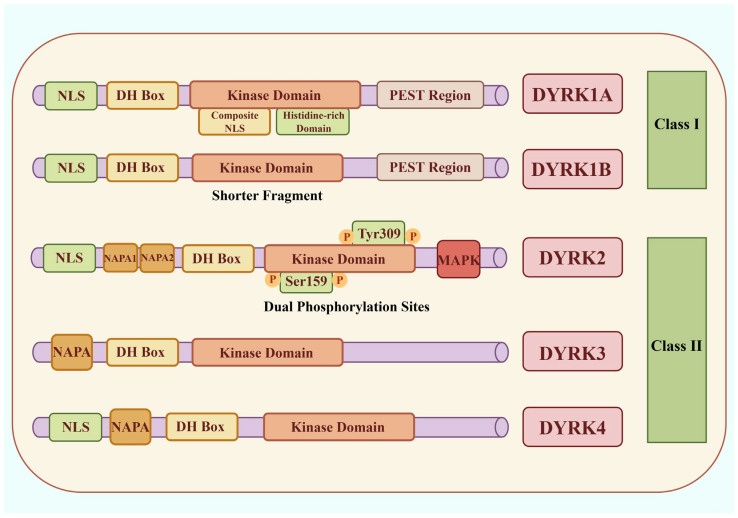
Structural Features of the DYRK Family. Class I DYRKs contain an NLS and a C-terminal PEST domain enriched in proline, glutamic acid, serine, and threonine residues, whereas Class II members harbor an NAPA domain. DYRK1A contains a bipartite N-terminal NLS and a second complex NLS within the catalytic domain. Compared with DYRK1A, DYRK1B has a shorter N-terminal region. DYRK2 contains two regulatory phosphorylation sites, Ser159 in the glycine-rich loop and Tyr309 in the activation loop. With the exception of DYRK3, all DYRK family members contain an NLS. NLS: Nuclear Localization Signal; DH Box: DEAD-box helicase domain; NAPA: N-terminal autophosphorylation accessory domain; PEST: Proline (P), Glutamic acid (E), Serine (S), Threonine (T) domain; MAPK: mitogen-activated protein kinase.

Dual-specificity tyrosine-regulated kinases (DYRKs) belong to the CMGC kinase family, which also includes cyclin-dependent kinases (CDKs), mitogen-activated protein kinases (MAPKs), glycogen synthase kinases (GSKs), and CDC-like kinases (CLKs) [9]. The human DYRK family consists of five members and is classified into class I (DYRK1A/1B) and class II (DYRK2-4) based on structural features and subcellular localization [10,11,12]. All DYRKs share a conserved catalytic kinase domain and an N-terminal DYRK homology (DH) box, which together form the structural basis for substrate recognition and catalysis [13,14] (Figure 1). A conserved activation-loop YxY motif is essential for kinase maturation [1].

Class I DYRKs contain nuclear localization signals and C-terminal PEST-rich regions, findings consistent with their roles in nuclear signaling, transcriptional regulation, and RNA processing. DYRK1A also contains a polyhistidine tract that targets it to nuclear speckles and regulates pre-messenger RNA (mRNA) splicing [15]. By contrast, class II DYRKs contain an N-terminal autophosphorylation accessory (NAPA) domain required for activation-loop tyrosine phosphorylation [16]. These kinases are mainly cytoplasmic but translocate to the nucleus in response to stress [3,17,18]. Notably, the N-terminus of DYRK3 mediates recruitment to stress granules, implicating this isoform in RNA metabolism and stress responses [19]. Single-cell transcriptomic analysis of the adult human heart shows distinct DYRK expression patterns [20]: DYRK1A and DYRK2 are broadly expressed across cardiomyocytes, fibroblasts, and endothelial cells, whereas DYRK1B, DYRK3, and DYRK4 exhibit weak transcriptional signals. These patterns suggest DYRK1A may be the dominant cardiac isoform in the heart, with other members’ roles yet to be elucidated.

### 2.2. DYRKs and Cell Cycle

Cell cycle regulation is essential for cardiac homeostasis and the response to injury [21]. In recent years, targeting the cell cycle to induce cardiomyocyte regeneration has emerged as a promising therapeutic strategy for cardiovascular diseases such as myocardial infarction and heart failure [22]. Given the limited regenerative capacity of the adult heart, elucidating the regulatory network of key cell cycle factors is crucial for developing cardiac repair strategies [23]. Among these factors, DYRK family has attracted attention because of its central role in phosphorylating cell cycle proteins [24].

Dyrk1a functions in a dosage-dependent manner. It phosphorylates cyclin D1 at Thr286, promoting its nuclear export and proteasomal degradation and thereby regulating the G1/S transition. This process influences the balance between cyclin D1 and p21, determining whether cells proliferate or undergo differentiation, quiescence, or senescence [25,26,27]. Dysregulated DYRK1A-mediated cell cycle control has been linked to dilated cardiomyopathy, heart failure, and congenital heart defects [28,29].

DYRK1B is mainly active in the G0/G1 phase. It phosphorylates cyclin D1 at Thr288 and cooperates with glycogen synthase kinase 3β (GSK3β) to promote its degradation, thereby inhibiting the G1/S transition. DYRK1B also phosphorylates p27 at Ser10, enhancing its stability and CDK-inhibitory activity, and thus reinforcing G0 arrest. In cancer, however, DYRK1B is often overexpressed and aberrantly activated, potentially altering p27 function to promote tumor progression, thus establishing it as a potential cancer therapeutic target [30,31,32]. Although its direct role in the injured heart remains unclear, these mechanisms suggest that DYRK1B may limit cardiomyocyte cell cycle re-entry after injury.

DYRK2 suppresses the G1/S transition by promoting the degradation of c-Jun and c-Myc. In the G1 phase, DYRK2 acts as a priming kinase for GSK-3β, phosphorylating c-Jun and c-Myc to target them for ubiquitin-proteasomal degradation, thereby suppressing excessive proliferation [33]. In the G2/M phase, it also facilitates the degradation of proteins such as Telomerase reverse transcriptase (TERT) and katanin p60 through the EDD-DDB1-VprBP (EDVP) E3 ligase complex, contributing to tumor-suppressive functions [34]. However, its role in the heart remains largely unknown.

In contrast, the roles of DYRK3 and DYRK4 in cell cycle regulation are less clear. DYRK3 appears to participate in mitotic progression, but its specific functions remain to be defined [35], whereas no direct evidence currently links DYRK4 to cell cycle control. Overall, the DYRK family represents a potential target for promoting cardiomyocyte regeneration and treating cardiac disease.

### 2.3. DYRKs and DNA Damage

DNA damage is a major contributor to cardiovascular disease progression. Unrepaired DNA lesions caused by endogenous metabolic stress or exogenous insults activate the DNA damage response (DDR), and persistent DDR activation promotes senescence, apoptosis, and inflammation, thereby impairing cardiac function and accelerating atherosclerosis, cardiomyopathy, and heart failure [36].

DYRK family kinases participate in DNA damage repair through multiple mechanisms. DYRK1A promotes homologous recombination, a high-fidelity pathway for DNA double-strand break (DSB) repair, by phosphorylating RNF169 at Ser368 and Ser403, which enhances its ability to antagonize 53BP1 accumulation at DSB sites [37]. In B-cell acute lymphoblastic leukemia, DYRK1A also phosphorylates Forkhead box protein O 1 (FOXO1), suppressing its nuclear activity and negatively regulating the DDR pathway [38]. DYRK1B contributes to the nucleolar DNA damage response by suppressing ribosomal DNA (rDNA) transcription in a kinase-dependent manner, thereby facilitating DSB repair and preserving rDNA stability [39]. In addition, DYRK1B is recruited to DSBs in a poly-ADP-ribose-polymerase (PARP)-dependent manner, where it phosphorylates the histone methyltransferase EHMT2 to inhibit local transcription and promote DNA repair [40].

DYRK2 serves as an important link between DNA damage and apoptosis. In response to severe or irreparable DNA damage, DYRK2 translocates to the nucleus downstream of ataxia telangiectasia Mutated (ATM) signaling and directly phosphorylates p53 at Ser46, thereby inducing pro-apoptotic gene expression and triggering apoptosis [41]. Through this mechanism, DYRK2 helps eliminate heavily damaged cells and preserve genomic stability. Overall, DYRKs are important regulators of DNA repair and genome integrity. In the cardiovascular system, their dysregulation may contribute to disease pathogenesis, further highlighting the DYRK family as a potential therapeutic target in cardiovascular disease.

### 2.4. DYRKs and Apoptosis

Apoptosis is a key contributor to the progression of cardiovascular diseases, including myocardial infarction, ischemia/reperfusion injury, heart failure, and diabetic cardiomyopathy [42]. Members of the DYRK family regulate apoptosis through distinct, context-dependent mechanisms. DYRK1A and DYRK3 exert anti-apoptotic effects by phosphorylating and activating SIRT1, an NAD+-dependent protein deacetylase, which enhances p53 deacetylation and suppresses p53-dependent apoptotic transcription under genotoxic stress [43]. Notably, DYRK1A has dual roles in apoptosis depending on the biological context. It suppresses apoptosis by phosphorylating caspase-9 during retinal development and promotes cell survival in leukemia through regulation of FOXO1 and signal transducer and activator of transcription 3 (STAT3), whereas in colorectal cancer and myocardial ischemia/reperfusion injury it may increase apoptotic sensitivity or promote ferroptosis [44,45,46,47].

The role of DYRK1B in apoptosis is likewise context-dependent. It generally inhibits apoptosis and promotes chemoresistance in cancer cells, but can also facilitate motor neuron apoptosis during embryonic development [48,49]. DYRK2, by contrast, promotes p53-dependent apoptosis through phosphorylation of p53 at Ser46. Under basal conditions, nuclear DYRK2 interacts with murine double minute 2 (MDM2) and undergoes ubiquitin-mediated proteasomal degradation. Following DNA damage, ATM phosphorylates DYRK2 at Thr33 and Ser369, disrupting its association with MDM2 and preventing its degradation. This results in nuclear accumulation and activation of DYRK2, which in turn phosphorylates p53 at Ser46 and initiates apoptosis [18,50].

In summary, under stress conditions such as myocardial ischemia–reperfusion, upregulation of DYRK1A correlates with increased cardiomyocyte death, potentially involving both apoptotic and ferroptotic pathways [47]. Conversely, DYRK1A and DYRK3 exert cardioprotective effects by activating the SIRT1-p53 axis to inhibit apoptosis [43]. In addition, DYRK2 may influence cardiomyocyte survival by modulating the ATM-MDM2-p53 pathway [18,50]. These findings identify DYRKs and their downstream signaling networks as potential therapeutic targets in cardiovascular disease.

### 2.5. DYRKs and Oxidative Stress

Oxidative stress is defined as an imbalance between pro-oxidants and antioxidants that disrupts redox signaling and homeostasis [51]. In the cardiovascular system, oxidative stress is a common pathogenic basis for the initiation and progression of multiple cardiovascular diseases [52]. Although physiological levels of reactive oxygen species (ROS) are essential for cellular function, excessive ROS damages DNA, lipids, and proteins, thereby promoting endothelial dysfunction, hypertension, heart failure, and atherosclerosis [53].

Current studies on DYRKs in oxidative stress mainly focus on DYRK1 class kinases. *Dyrk1a* exerts antioxidant effects by activating ERK1/2, promoting Nrf2 nuclear translocation, and upregulating NAD(P)H quinone dehydrogenase 1 (NQO1) expression [54]. DYRK1B likewise appears to suppress ROS accumulation, as its inhibition or knockdown in C2C12 cells increases intracellular ROS levels. This effect may involve maintenance of pyruvate dehydrogenase complex activity and induction of antioxidant gene expression [55,56]. Collectively, DYRK1 kinases may serve as potential therapeutic targets for oxidative stress-related cardiovascular disease.

## 3. DYRKs and Cardiovascular Disease

### 3.1. Myocardial Infarction

Myocardial infarction (MI) is characterized by cardiomyocyte necrosis caused by prolonged ischemia and hypoxia due to a marked reduction or interruption of coronary blood flow, most commonly resulting from coronary atherosclerosis [57]. Because adult mammalian cardiomyocytes have limited regenerative capacity after MI, stimulating their endogenous proliferation has emerged as a promising strategy for cardiac repair [2].

DYRK1A has been identified as a key suppressor of cardiomyocyte cell-cycle re-entry. Mechanistically, DYRK1A phosphorylates LIN52 at Ser28, thereby promoting DREAM (DP, RB [retinoblastoma], E2F, and MuvB) complex assembly and repressing the transcription of genes required for the G1/S and G2/M transitions [58]. DYRK1A also phosphorylates WDR82, a component of the H3K4me3 methyltransferase complex, and KAT6A, an H3K27 acetyltransferase, thereby reducing histone 3 lysine 4 trimethylation (H3K4me3) and histone 3 lysine 27 acetylation (H3K27ac) deposition at the promoters of cell-cycle genes and suppressing proliferation-associated transcription through epigenetic regulation [59].

**Figure 2:**
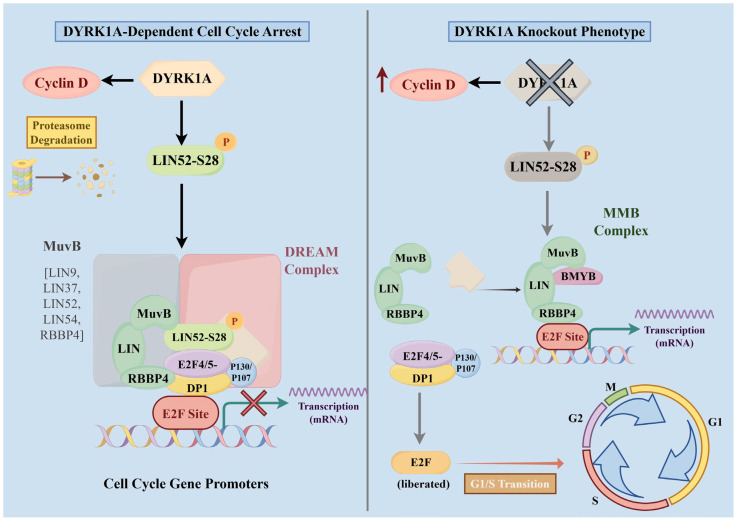
DYRK1A regulates the cardiomyocyte cell cycle following myocardial infarction. DYRK1A inhibits cell-cycle progression by promoting Cyclin D degradation and LIN52 Ser28 phosphorylation, thereby stabilizing the DREAM complex. DYRK1A deficiency elevates Cyclin D levels, disrupts DREAM assembly, promotes MMB complex formation and E2F release, and drives G1/S transition and cardiomyocyte proliferation. MuvB: Multi-vulval class B; DREAM complex: DP, RB-like, E2F, and MuvB complex; BMYB: MYB proto-oncogene like 2; MMB complex: Myb-MuvB complex.

In animal models, pharmacological inhibition of DYRK1A significantly increased cardiomyocyte cell-cycle re-entry after MI and improved left ventricular ejection fraction. Consistently, cardiomyocyte-specific deletion of *Dyrk1a* induced basal cardiomyocyte hyperplasia, improved post-MI cardiac function, and was associated with significant enrichment of mitotic cell-cycle genes [2]. Together, these findings indicate that DYRK1A restricts cardiomyocyte proliferation through both DREAM complex-dependent and epigenetic mechanisms. Accordingly, DYRK1A inhibition may promote myocardial repair and represents a potential therapeutic target for cardiac regeneration after MI (Figure 2).

### 3.2. Cardiac Hypertrophy

Cardiac hypertrophy is an adaptive response of the heart to chronic pressure or volume overload. While compensatory in its early stages, sustained pathological hypertrophy ultimately leads to heart failure and arrhythmias [60]. Its development is driven primarily by increased protein synthesis [61]. DYRKs have emerged as important regulators of this process, although their effects appear to be isoform-specific and context-dependent.

**Figure 3:**
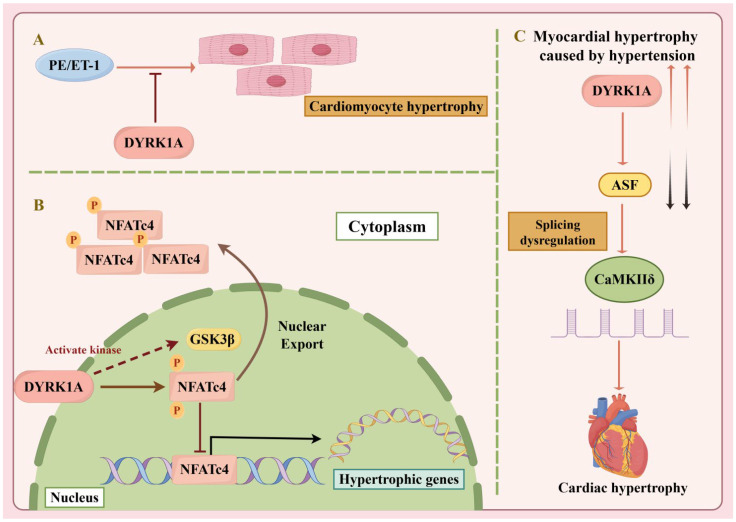
DYRK1A-mediated Regulation of Cardiac Hypertrophy. (**A**) DYRK1A opposes PE- and ET-1-induced cardiomyocyte hypertrophy. (**B**) DYRK1A phosphorylates NFAT and cooperates with GSK3β to promote NFAT nuclear export, thereby suppressing hypertrophic gene expression. (**C**) Dysregulation of the DYRK1A-ASF-CaMKIIδ pathway contributes to hypertension-induced cardiac hypertrophy. NFAT: Nuclear Factor of Activated T cells; GSK3β: Glycogen Synthase Kinase 3 β; ASF: Alternative Splicing Factor; CaMKIIδ: Calmodulin-dependent Protein Kinase IIδ.

DYRK1A generally acts as a negative regulator of hypertrophy, mainly by antagonizing the calcineurin-nuclear factor of activated T-cells (NFAT) pathway. DYRK1A phosphorylates NFAT transcription factors, promoting their nuclear export and thereby suppressing hypertrophic gene expression (Figure 3B) [62]. In vitro, DYRK1A overexpression attenuates agonist-induced cardiomyocyte hypertrophy. However, its protective role in vivo remains uncertain. In animal models, sustained cardiac *Dyrk1a* overexpression does not attenuate pressure overload-induced hypertrophy, suggesting compensatory mechanisms or limited activity in the physiological setting [63]. Moreover, in patients with aortic stenosis, elevated myocardial DYRK1A levels are associated with incomplete reverse remodeling and diastolic dysfunction, suggesting that DYRK1A may become maladaptive under chronic stress (Figure 3A) [64]. *Dyrk1a* also modulates hypertrophy through alternative pathways, including regulation of the splicing factor ASF and subsequent alternative splicing of Ca^2+^/calmodulin-dependent protein kinase IIδ (CaMKIIδ), thereby shifting expression from pro-hypertrophic to protective isoforms (Figure 3C) [65].

DYRK2 is another key negative regulator of cardiomyocyte growth [3]. As a priming kinase for GSK3β, DYRK2 phosphorylates eukaryotic translation initiation factor 2Bε (eIF2Bε) and facilitates its subsequent phosphorylation at Ser535 by GSK3β. This inhibits eIF2Bε GDP/GTP exchange activity, suppresses translation initiation, and thereby restrains the protein synthesis that drives hypertrophy [66]. However, under chronic pathological stimulation, the expression of DYRK2 cannot be sustained, leading to the failure of this inhibitory pathway and uncontrolled protein synthesis [67].

Together, these findings identify DYRK1A and DYRK2 as potential therapeutic targets for pathological cardiac hypertrophy. However, major challenges remain, including the context-dependent effects of DYRK1A in vivo and the loss of sustained DYRK2 expression during chronic stress. Based on the above mechanisms, we propose a therapeutic strategy for patients with hypertrophic cardiomyopathy that concurrently enhances DYRK1A-mediated NFAT nuclear export and DYRK2-mediated translational repression. This can be achieved by using small-molecule activators that co-activate both kinases. By leveraging the endogenous anti-hypertrophic functions of DYRK1A and DYRK2, this strategy addresses the potential issues of compensatory failure or chronic loss of expression associated with single-target approaches, offering a clear combinatorial path for clinical translation.

### 3.3. Myocardial Fibrosis

Myocardial fibrosis is characterized by excessive extracellular matrix deposition, resulting in ventricular stiffness and diastolic dysfunction [68]. DYRK1A has a dual role in this process. By limiting cardiomyocyte proliferation, DYRK1A may indirectly promote scar formation, whereas its deletion or inhibition enhances myocardial regeneration and reduces fibrosis [59]. Conversely, DYRK1A also suppresses fibrosis by phosphorylating NFAT and inhibiting calcineurin-NFAT signaling. After myocardial infarction, miR-199b-5p-mediated downregulation of *Dyrk1a* relieves this inhibition and aggravates fibrosis [69]. Transient receptor potential ankyrin 1 (TRPA1) activation in cardiac fibroblasts may further enhance calcineurin-NFAT signaling by suppressing DYRK1A and promoting Ca^2+^ influx [4].

**Figure 4:**
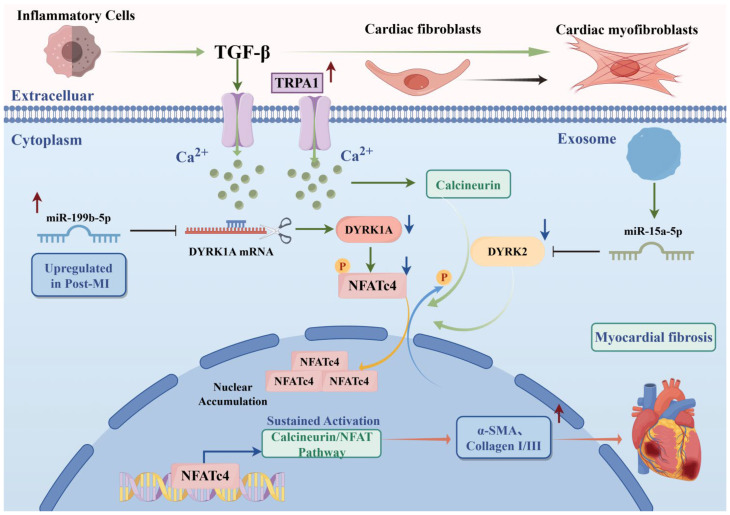
DYRK1A and DYRK2 regulate myocardial fibrosis. Inflammatory cell-derived TGF-β activates TRPA1, triggering Ca^2+^ influx and calcineurin activation. Cardiomyocyte-derived exosomal miR-199b-5p and miR-15a-5p suppress DYRK1A and DYRK2, respectively, leading to NFATc4 dephosphorylation and nuclear activation, induction of α-SMA and collagen I/III expression, and promotion of myocardial fibrosis. TGF-β: Transforming Growth Factor-β; NFATc4: Nuclear Factor of Activated T cells, cytoplasmic 4; TRPA1: Transient Receptor Potential Ankyrin 1; α-SMA: alpha-Smooth Muscle Actin.

DYRK2 has also been identified as a direct antifibrotic factor. By phosphorylating NFAT, *Dyrk2* suppresses its activity. Cardiomyocyte-derived exosomal miR-15a-5p targets *Dyrk2*, leading to NFAT dephosphorylation, nuclear translocation, and activation, which in turn promotes fibroblast activation and excessive collagen deposition. Conversely, *Dyrk2* overexpression or miR-15a-5p inhibition reverses the fibrotic phenotype [70]. Collectively, these findings identify DYRK1A and DYRK2 as potential therapeutic targets for myocardial fibrosis through direct or indirect modulation of pro-fibrotic signaling pathways (Figure 4).

### 3.4. Heart Failure

Heart failure (HF) is the end stage of many cardiac diseases [71]. Owing to its high energy demand, the heart is highly sensitive to impaired energy supply, and mitochondrial dysfunction is central to HF pathogenesis [72]. Abnormal activation of DYRK1A serves as one of the key mechanisms driving heart failure. Cardiac-specific overexpression of *Dyrk1a* directly leads to dilated cardiomyopathy and heart failure. The mechanism involves the phosphorylation and degradation of D-cyclins (e.g., Ccnd2), thereby inhibiting the Rb/E2F signaling pathway and impeding cardiomyocyte proliferation [28]. Furthermore, within the pathological context following myocardial infarction (MI), the activity and expression of *Dyrk1a* are enhanced. It disrupts the alternative splicing of CaMKIIδ by phosphorylating the splicing factor ASF, thereby driving pathological cardiac remodeling and the progression of heart failure; inhibition of *Dyrk1a* activity can ameliorate heart failure symptoms [73].

DYRK1B also plays a critical role in the pathogenesis of heart failure. Clinical studies have demonstrated that the transcriptional and protein levels of DYRK1B are significantly elevated in myocardial tissues from heart failure patients caused by dilated (DCM), ischemic (ICM), and hypertrophic (HCM) cardiomyopathy [74]. DYRK1B is primarily localized to the nucleus, and its nuclear abundance increases further following the impairment of cardiac function, suggesting its potential role in regulating nuclear transcriptional processes [75]. The specific mechanism involves the direct binding of DYRK1B, via its PEST domain, to the coiled-coil domain (CCD) of STAT3. This binding triggers the phosphorylation of STAT3 at Tyr705, promoting STAT3 dimerization and translocation into the nucleus. The activated and nuclear-accumulated STAT3 acts as a transcriptional repressor, directly negatively regulating the expression of peroxisome proliferator-activated receptor gamma coactivator 1-alpha (PGC-1α). PGC-1α is the master transcriptional coactivator controlling mitochondrial biogenesis and function. Its downregulation directly leads to impairments in mitochondrial bioenergetics, resulting in reduced mitochondrial number, dysfunction of the respiratory chain, decreased activity of electron transport chain (ETC) complexes, and impaired ATP synthesis. This directly exacerbates the core energy crisis in HF [74]. Overall, DYRK1A and DYRK1B represent potential therapeutic targets in HF, with the DYRK1B-STAT3-PGC-1α pathway being particularly attractive for restoring mitochondrial function.

### 3.5. Myocardial Ischemia–Reperfusion Injury

Myocardial ischemia–reperfusion injury (MIRI) is a paradoxical pathological process in which restoration of blood flow after transient ischemia further aggravates myocardial damage and dysfunction. The underlying mechanisms include oxidative stress, calcium overload, inflammatory activation, and multiple forms of regulated cell death [76]. Among these, ferroptosis, an iron-dependent form of regulated cell death, plays a central role in MIRI. Ferroptosis is characterized by collapse of the intracellular antioxidant defense system, particularly dysfunction of the glutathione-GPX4 axis, leading to excessive lipid peroxide accumulation, loss of membrane integrity, and ultimately cell death [77].

Recent studies have shown that DYRK1A expression is significantly upregulated during MIRI and exacerbates cardiac injury by promoting ferroptosis. Mechanistically, DYRK1A increases the expression of the pro-ferroptotic proteins Acyl-CoA synthetase long-chain family member 4 (ACSL4) and Transferrin receptor 1 (TFR1) while suppressing the antiferroptotic protein Glutathione peroxidase 4 (GPX4) and its associated transporter Solute carrier family 7 member 11 (SLC7A11). These changes aggravate lipid peroxidation and iron dysregulation, thereby inducing cardiomyocyte ferroptosis. Conversely, *Dyrk1a* knockdown reverses these molecular changes and attenuates myocardial injury. Moreover, the protective effect of *Dyrk1a* knockdown is abolished by the ferroptosis inducer erastin, further supporting the conclusion that DYRK1A aggravates MIRI through ferroptosis [47]. Therefore, targeting DYRK1A may represent a promising cardioprotective strategy.

### 3.6. Down Syndrome-Associated Congenital Heart Disease

Down syndrome-associated congenital heart disease (DS-CHD) comprises a group of common structural cardiac abnormalities associated with trisomy 21. It arises from disruption of embryonic cardiac developmental programs caused by chromosome dosage imbalance, with atrioventricular septal defects being among the most typical phenotypes [78]. Research indicates that the *Dyrk1a* gene located on chromosome 21 is a central pathogenic gene for DS-CHD. Its increased dosage (triploid) contributes to abnormal heart development through multiple mechanisms [29].

At the cellular proliferation level, DYRK1A impedes cell cycle progression by phosphorylating key factors such as cyclin D and LIN52 [58]. In terms of mitochondrial function, overexpression of *Dyrk1a* compromises mitochondrial respiratory efficiency and oxidative phosphorylation capacity [79]. DYRK1A, whose Tyr321 phosphorylation is increased in Down syndrome hearts, also phosphorylates the splicing factor SRSF6, thereby altering the alternative splicing of genes such as cardiac troponin T (TNNT2) and disrupting the balance of contractile protein isoforms [80]. Notably, these abnormal phenotypes can be reversed when *Dyrk1a* gene dosage is restored to normal, and the use of highly specific inhibitors can partially restore the expression of related genes [81]. These findings suggest that DYRK1A is a potential therapeutic target for DS-CHD.

### 3.7. Other

Gain-of-function mutations in the *DYRK1B* gene are a direct cause of familial early-onset metabolic syndrome, with central obesity and insulin resistance as its core features. Normal *Dyrk1b* promotes adipogenesis by inhibiting the SHH signaling pathway and accelerating p27Kip degradation; the mutant form enhances these effects and additionally promotes the expression of the rate-limiting gluconeogenic enzyme G6Pase, thereby exacerbating obesity and hyperglycemia [82]. At the molecular level, this mutation directly activates the mammalian Target of Rapamycin Complex 2 (mTORC2) complex, upregulates lipogenic enzymes, increases fatty acid uptake, and promotes hepatic fat accumulation [5]. Concurrently, it enhances the nuclear retention and activity of the transcription factor FOXO1 through phosphorylation, leading to increased expression of key gluconeogenic enzymes and promoting hepatic glucose output. Furthermore, the excessive inhibition of the RAS-RAF-MEK pathway by mutant *Dyrk1b* aggravates insulin resistance. Additionally, the lipid accumulation mediated by *Dyrk1b* activates Protein Kinase C ε (PKCε), which phosphorylates and inhibits the insulin receptor, worsening glucose metabolism [83]. These alterations collectively drive central obesity and insulin resistance, which in turn form a critical pathological basis for hypertension and atherosclerosis, ultimately leading to cardiovascular complications such as coronary heart disease.

## 4. DYRK Inhibitors and Pharmacotherapy

Given the involvement of DYRKs in multiple pathological processes underlying cardiovascular disease, these kinases have attracted increasing interest as potential therapeutic targets, although their cardiovascular-specific mechanisms remain incompletely understood [84,85,86]. To date, inhibitors targeting DYRKs have shown encouraging progress in cancer and neurological disorders [87,88,89], whereas studies in cardiovascular disease remain largely preclinical (Table 1). Notably, no DYRK inhibitor has yet entered clinical trials specifically for cardiovascular indications [90].

Among DYRK family members, cardiovascular research has focused primarily on DYRK1A because of its central roles in cardiomyocyte cell cycle regulation, cardiac development, and injury repair. As DYRK1A inhibitors have been comprehensively reviewed in other disease contexts, this section highlights representative studies in cardiovascular pathology [81,92,93,94]. Several small-molecule DYRK1A inhibitors, including harmine, leucettine-21, leucettine-92, and epigallocatechin gallate (EGCG), have shown cardioprotective effects in cellular and animal models. These effects involve multiple mechanisms, including modulation of the calcineurin/NFAT pathway, regulation of ASF-dependent alternative splicing of CaMKIIδ, and epigenetic control of cardiac developmental gene expression through histone phosphorylation [2,59,73].

Among these compounds, harmine was one of the earliest and most extensively studied DYRK1A inhibitors; however, its concurrent inhibition of monoamine oxidase A (MAO-A) raises neurotoxicity concerns and limits its clinical translation. By contrast, second-generation inhibitors such as leucettine-21 and leucettine-92 show improved selectivity while retaining potent DYRK1A inhibitory activity. Notably, leucettine-21 partially rescues congenital cardiac abnormalities caused by *Dyrk1a* gene dosage imbalance in Down syndrome models, suggesting potential translational value in developmental heart disease [29]. Consistent with pharmacological inhibition, genetic silencing of *Dyrk1a* by short hairpin RNA (shRNA) or small interfering RNA (siRNA) also attenuates ischemia/reperfusion injury by suppressing cardiomyocyte ferroptosis and promotes cardiomyocyte proliferation and functional recovery after myocardial infarction [2,47].

In contrast, pharmacological studies targeting DYRK1B and other DYRK family members remain limited and are used mainly as tools for mechanistic investigation [95]. DYRK1B contributes to maintenance of cardiomyocyte quiescence and is activated under oxidative stress. The DYRK1B inhibitor AZ191 has been shown to attenuate pathological cardiac hypertrophy and heart failure phenotypes in cellular and animal models by blocking DYRK1B-STAT3 signaling and restoring PGC-1α expression and mitochondrial bioenergetic function. Although DYRK1B is expressed at lower levels than DYRK1A in cardiomyocytes, its stress-induced upregulation suggests a specific role in maladaptive cardiac remodeling [74].

For DYRK2, current cardiovascular evidence is derived largely from mechanistic studies. DYRK2 is broadly expressed in the heart and can be activated by DNA damage and oxidative stress, thereby participating in p53-dependent apoptotic signaling. The small-molecule inhibitor C17 modulates calcium influx by inhibiting stromal interaction molecule 1 (STIM1) phosphorylation, suggesting potential antiarrhythmic activity; however, its systemic effects in cardiovascular disease models remain unclear [96,97]. DYRK2 also phosphorylates multiple translation elongation factors and may therefore contribute to cardiomyocyte protein synthesis and pathological hypertrophy, although this requires validation in vivo and in clinical samples [98,99].

Research on DYRK3 and DYRK4 in the cardiovascular system remains sparse. DYRK3 is mainly expressed in hematopoietic tissues but is also detectable in the heart [100], whereas DYRK4 is expressed at very low levels in cardiac tissue and has not been directly studied in cardiovascular contexts. Accordingly, most available studies still rely on pan-DYRK inhibitors, while the development of highly selective modulators for DYRK3 or DYRK4 remains underdeveloped [101].

Translation of DYRK-targeted therapies to cardiovascular disease will require subtype-specific strategies. For DYRK1A, the major challenge is to balance regenerative benefit against potential tumorigenic risk, highlighting the need for cardiomyocyte-targeted delivery and improved second-generation inhibitors [7,59,73]. For DYRK1B, progress is constrained by the lack of highly selective compounds; drug repurposing and evaluation in metabolically high-risk populations may therefore be practical approaches [102,103]. For DYRK2, priority should be given to establishing pharmacodynamic biomarkers such as STIM1 and p53 and to evaluating short-term intervention strategies that may limit long-term adverse effects [96]. On this basis, future clinical studies may focus on acute myocardial infarction or Down syndrome-associated congenital heart disease for DYRK1A, HFpEF with obesity or diabetes for DYRK1B, and perioperative prevention of post-surgical atrial fibrillation for DYRK2.

## 5. Discussion

The DYRK family has emerged as an important molecular hub in the regulation of cardiovascular homeostasis and pathological remodeling. Through their distinctive auto-activation mechanism, DYRKs integrate signaling pathways that govern key biological processes, including cardiomyocyte cell-cycle control, apoptosis, the DNA damage response, and oxidative stress. Accumulating evidence has increasingly clarified the molecular basis by which DYRKs contribute to diverse cardiovascular pathologies, including impaired myocardial regeneration, pathological hypertrophy, fibrosis, and ischemia/reperfusion injury. Collectively, these findings suggest that DYRKs may function at the intersection of myocardial regenerative capacity, metabolic adaptation, and structural remodeling.

Despite these advances, the roles of DYRKs in the cardiovascular system remain incompletely understood and, in some cases, controversial. Most available evidence is derived from cardiomyocyte-based systems or acute murine injury models, whereas the functions of DYRK signaling in other major cardiac cell types, such as fibroblasts, endothelial cells, and immune cells, remain poorly defined. Moreover, these experimental models do not fully recapitulate the complex and prolonged progression of chronic human cardiovascular disease. The functional divergence among DYRK family members also remains unclear. In particular, the physiological and pathological roles of DYRK3 and DYRK4 in the heart are largely unexplored, and whether distinct isoforms exhibit functional redundancy or coordinated regulation requires further investigation.

Notably, several studies have yielded apparently inconsistent results. For example, in pressure overload-induced cardiac hypertrophy, acute in vitro models indicate that DYRK1A exerts a marked inhibitory effect. However, in chronic in vivo settings, *Dyrk1a* overexpression does not substantially prevent hypertrophic remodeling, and elevated *DYRK1A* expression in the reverse-remodeling phase of human disease has been associated with worse prognosis. In addition, the function of DYRK1A may vary across cell types. In cardiomyocytes, DYRK1A inhibition may indirectly exert antifibrotic effects by promoting cell-cycle re-entry, whereas in fibroblasts, DYRK1A participates more directly in profibrotic signaling, including NFAT-related pathways. Consequently, DYRK1A inhibition may produce divergent, or even opposing, biological effects depending on the cellular and pathological context. These observations underscore the highly context-dependent nature of DYRK family function, particularly that of DYRK1A, which is shaped by cell type, disease stage, and the activity of downstream signaling networks.

From a translational perspective, the development of DYRK-targeted therapies still faces several major challenges. Currently available DYRK inhibitors generally show limited isoform selectivity, raising the possibility of cross-kinase inhibition and other off-target effects. Long-term systemic inhibition may also pose safety concerns, particularly with respect to the central nervous system and potential tumor-promoting risk. These issues require rigorous evaluation in future preclinical and clinical studies. In addition, because DYRK signaling may exert stage-specific and cell type-specific effects, the optimal therapeutic window and criteria for patient stratification remain to be established.

For example, DYRK1A is known to be a negative regulator of the cell cycle; its inhibition promotes cardiomyocyte proliferation and functional recovery after myocardial infarction, whereas complete and sustained cardiac DYRK1A loss may theoretically lead to hyperproliferation. Thus, a temporal strategy—transient inhibition during the acute repair phase followed by restoration of DYRK1A activity after healing—is critical for achieving a safe therapeutic window. This strategy remains conceptual, and future studies in chronic disease models are required to define the permissible depth and duration of DYRK1A inhibition to avoid adverse structural and electrophysiological remodeling.

In addressing the above challenges, significant progress has been made in cardiac-specific delivery technologies. Engineered AAV vectors incorporating capsid modifications, cardiac-specific promoters, and microRNA target sequences enable selective cardiac gene editing while reducing hepatic off-target effects [104,105]. Lipid nanoparticles (LNPs) combined with intracoronary injection achieve efficient mRNA delivery in myocardial ischemia–reperfusion models [106]. Self-amplifying RNA (saRNA) technology is particularly notable: in myocardial infarction models, a single low dose achieves robust and sustained protein expression with minimal off-target expression in extracardiac tissues [107]. Integrating these delivery platforms with DYRK-targeted strategies—such as intracoronary LNP delivery of DYRK1A-inhibitory saRNA in acute ischemia, or cardiac-specific AAV-mediated DYRK1B gene silencing in chronic heart failure—may enable spatiotemporally controllable, isoform-selective precision therapies, providing a key technological foundation for the clinical translation of DYRK-targeted therapeutics.

Future investigations should integrate advanced approaches, including single-cell omics, multi-omics analysis, and structural biology, to systematically define the dynamic regulatory networks of DYRKs across cardiac cell populations and disease stages. Such efforts will also help elucidate the structural determinants of isoform-specific function and facilitate the development of more selective therapeutic strategies. In parallel, allosteric approaches targeting non-catalytic domains or protein–protein interaction interfaces should be explored, particularly in combination with cardiac-targeted delivery systems to minimize systemic exposure. Therefore, a systematic understanding of the context-dependent regulation of DYRK family members across cardiac cell types and disease stages will be essential for advancing the clinical translation of DYRK-targeted therapies.

## Figures and Tables

**Table 1:** Advances in DYRK family kinase inhibitors for cardiovascular diseases.

DYRK Inhibitor	Primary Target	Model System	Core Mechanism	Development Stage	Cardiovascular Effect/Potential	Refs.
Harmine	DYRK1A	In vitro, In vivo (mouse MI/I-R models)	Inhibition of DYRK1A-ASF-CaMKIIδ and Calcinerun-NFAT signaling; epigenetic regulation of cardiomyocyte cell cycle	Preclinical	Promotes cardiomyocyte proliferation; attenuates myocardial fibrosis and hypertrophy; improves cardiac function after MI or ischemia–reperfusion injury	[2,59,73]
Leucettinib-21	DYRK1A	In vivo (mouse)	Partial normalization of *Dyrk1a* dosage–dependent transcriptional programs; enhancement of cardiomyocyte proliferative capacity	Preclinical	Ameliorates congenital heart defects associated with *DYRK1A* overexpression	[29]
Leucettinib-92	DYRK1A	In vivo (mouse MI model)	Activation of cardiomyocyte cell cycle through DYRK1A inhibition	Preclinical	Improves cardiac function following acute myocardial infarction	[91]
EGCG	DYRK1A	In vitro; In vivo (rat HF models)	Modulation of DYRK1A-ASF-CaMKIIδ signaling and alternative splicing	Preclinical	Attenuates pathological cardiac hypertrophy and delays heart failure progression	[73]
AZ191	DYRK1B	In vitro; In vivo (mouse HF models)	Inhibition of DYRK1B-STAT3 axis, restoration of PGC-1α-dependent mitochondrial bioenergetics	Preclinical	Alleviates pathological cardiac hypertrophy and improves cardiac function in heart failure	[74]

## Data Availability

No new data were created or analyzed in this study. Data sharing is not applicable to this article.

## Abbreviations

The following abbreviations are used in this manuscript:

α-SMA	alpha-Smooth Muscle Actin
ACSL4	Acyl-CoA synthetase long-chain family member 4
ASF	Alternative Splicing Factor
ATM	Ataxia telangiectasia Mutated
BMYB	MYB proto-oncogene like 2
CaMKIIδ	Ca^2+^/calmodulin-dependent protein kinase IIδ
CCD	Coiled-coil domain
CDKs	Cyclin-dependent kinases
CLKs	CDC-like kinases
CVDs	Cardiovascular diseases
DCM	Dilated cardiomyopathy
DDR	DNA damage response
DH	DYRK homology
DREAM	DP, RB [retinoblastoma], E2F, and MuvB
DSB	DNA double-strand break
DS-CHD	Down syndrome-associated congenital heart disease
DYRK	Dual-specificity tyrosine-regulated kinase
EDVP	EDD-DDB1-VprBP
EGCG	Epigallocatechin gallate
eIF2Bε	Eukaryotic translation initiation factor 2Bε
ETC	Electron transport chain
FOXO1	Forkhead box protein O 1
GPX4	Glutathione peroxidase 4
GSK3β	Glycogen synthase kinase 3β
GSKs	Glycogen synthase kinases
HCM	Hypertrophic cardiomyopathy
HF	Heart failure
ICM	Ischemic cardiomyopathy
MAO-A	Monoamine oxidase A
MAPKs	Mitogen-activated protein kinases
MDM2	Murine double minute 2
MI	Myocardial infarction
MIRI	Myocardial ischemia–reperfusion injury
MMB	Myb-MuvB
mTORC2	mammalian Target of Rapamycin Complex 2
MuvB	Multi-vulval class B
NAPA	N-terminal autophosphorylation accessory
NFATc4	Nuclear factor of activated T-cells, cytoplasmic 4
NLS	Nuclear Localization Signal
NQO1	NAD(P)H quinone dehydrogenase 1
PARP	Poly-ADP-ribose-polymerase
PEST	Proline (P), Glutamic acid (E), Serine (S), Threonine (T) domain
PGC-1α	Peroxisome proliferator-activated receptor gamma coactivator 1-alpha
PKCε	Protein Kinase C ε
rDNA	Ribosomal DNA
ROS	reactive oxygen species
SLC7A11	Solute carrier family 7 member 11
STAT3	Signal transducer and activator of transcription 3
STIM1	Stromal interaction molecule 1
TERT	Telomerase reverse transcriptase
TFR1	Transferrin receptor 1
TGF-β	Transforming Growth Factor-β
TRPA1	Transient Receptor Potential Ankyrin 1
LNPs	Lipid nanoparticles
saRNA	Self-amplifying RNA
